# Genomic prediction of hybrid performance for agronomic traits in sorghum

**DOI:** 10.1093/g3journal/jkac311

**Published:** 2022-12-01

**Authors:** Sirjan Sapkota, Jon Lucas Boatwright, Neeraj Kumar, Matthew Myers, Alex Cox, Arlyn Ackerman, William Caughman, Zachary W Brenton, Richard E Boyles, Stephen Kresovich

**Affiliations:** Advanced Plant Technology Program, Clemson University, Clemson, SC 29634, USA; Advanced Plant Technology Program, Clemson University, Clemson, SC 29634, USA; Department of Plant and Environmental Sciences, Clemson University, Clemson, SC 29634, USA; Advanced Plant Technology Program, Clemson University, Clemson, SC 29634, USA; Department of Plant and Environmental Sciences, Clemson University, Clemson, SC 29634, USA; Advanced Plant Technology Program, Clemson University, Clemson, SC 29634, USA; Advanced Plant Technology Program, Clemson University, Clemson, SC 29634, USA; Department of Plant and Environmental Sciences, Clemson University, Clemson, SC 29634, USA; Pee Dee Research and Education Center, Clemson University, Florence, SC 29506, USA; Pee Dee Research and Education Center, Clemson University, Florence, SC 29506, USA; Carolina Seed Systems, Inc., Florence, SC 29506, USA; Department of Plant and Environmental Sciences, Clemson University, Clemson, SC 29634, USA; Pee Dee Research and Education Center, Clemson University, Florence, SC 29506, USA; Advanced Plant Technology Program, Clemson University, Clemson, SC 29634, USA; Department of Plant and Environmental Sciences, Clemson University, Clemson, SC 29634, USA

**Keywords:** genomic selection, heterosis, training population, cereal crop, hybrid breeding, GBLUP

## Abstract

Hybrid breeding in sorghum [*Sorghum bicolor* (L.) Moench] utilizes the cytoplasmic-nuclear male sterility (CMS) system for seed production and subsequently harnesses heterosis. Since the cost of developing and evaluating inbred and hybrid lines in the CMS system is costly and time-consuming, genomic prediction of parental lines and hybrids is based on genetic data genotype. We generated 602 hybrids by crossing two female (A) lines with 301 diverse and elite male (R) lines from the sorghum association panel and collected phenotypic data for agronomic traits over two years. We genotyped the inbred parents using whole genome resequencing and used 2,687,342 high quality (minor allele frequency > 2%) single nucleotide polymorphisms for genomic prediction. For grain yield, the experimental hybrids exhibited an average mid-parent heterosis of 40%. Genomic best linear unbiased prediction (GBLUP) for hybrid performance yielded an average prediction accuracy of 0.76–0.93 under the prediction scenario where both parental lines in validation sets were included in the training sets (T2). However, when only female tester was shared between training and validation sets (T1F), prediction accuracies declined by 12–90%, with plant height showing the greatest decline. Mean accuracies for predicting the general combining ability of male parents ranged from 0.33 to 0.62 for all traits. Our results showed hybrid performance for agronomic traits can be predicted with high accuracy, and optimizing genomic relationship is essential for optimal training population design for genomic selection in sorghum breeding.

## Introduction

Heterosis is the phenomenon by which the $F_{1}$ progeny of inbred lines outperform their parental lines for desired traits of interest (Birchler *et al.* 2010). It was first discovered and implemented in maize breeding by Shull and East, and by the middle of the twentieth century heterosis was being harnessed across multiple crop species in the form of hybrid cultivars (Shull 1908; East 1909). Mid-parent heterosis (MPH) is the difference between genetic value of $F_{1}$ progeny and average of its parents’ genetic values (Lynch and Walsh 1998). The genetic value of a progeny is expected to be equal to the average genetic value of its parents under the additive gene action model. Therefore, MPH results from dominance and epistatic deviation (Labroo, Studer, *et al.* 2021). However, it is important to define heterosis at the population level because breeding and selection happens at the population level. When parental populations are inbred to homozygosity, the inbred-midparent heterosis (IMPH) is defined as the difference between the mean of the $F_{1}$ and the mean of the parent populations (Lamkey and Edwards 1999). IMPH arises from the baseline heterosis, which is the lost vigor during inbreeding, plus the panmictic-midparent heterosis, which is observed when two random mating populations are crossed to form an $F_{1}$ hybrid (Lamkey and Edwards 1999). Therefore, IMPH is a function of inbreeding depression, genetic divergence, and dominance whereas panmictic-midparent heterosis is a function only of genetic divergence and dominance (Lamkey and Edwards 1999).

In maize, heterosis is exploited by creating single-cross hybrids from two genetically distinct heterotic pools. The distinct heterotic groups in maize were developed from individuals that shared nearly half of their genetic material which were then deliberately isolated and bred to harness the heterotic advantage (Tracy and Chandler 2006). While it was easier to make crosses and produce hybrid seeds in commercial capacity in maize due to separation of the male and female flowers, the same feat was arduous in self-pollinated species until the discovery of male sterility systems (Duvick 1959). Sorghum [*Sorghum bicolor* (L.) Moench] is one of the self-pollinated crops that is bred as a hybrid crop, particularly in the United States (US) and Australia. Beginning in the 1950s, sorghum breeding in the US started utilizing the cytoplasmic-nuclear male sterility (CMS) system to produce hybrid cultivars (Stephens and Holland 1954). The CMS system uses the three lines system which includes male sterile female lines, maintainers of the female lines that are genetically identical but without male sterile cytoplasm, and restorer lines that have genes that are capable of restoring the fertility of the male sterile lines, commonly referred to as the A, B, and R lines, respectively (Bohra *et al.* 2016). While A and R populations in sorghum breeding are utilized in the same manner as heterotic pools in maize, the two pools may not be genetic diverged into clear heterotic groups. While producing hybrid seeds from the A and R line crosses are convenient, the process of developing inbreds, particularly the pairs of A/B parental lines, is laborious and time-consuming because of the need for introgression of cytoplasm and backcrossing (Crozier *et al.* 2020).

The primary objective in hybrid breeding is inter-population improvement by reciprocal recurrent selection of individuals based on their performance as parents in between-population crosses and involves not only the improvement of parental lines themselves but also the performance of their hybrid combinations (Hallauer *et al.* 2010). Therefore, the two major goals in hybrid breeding program are: (1) to cyclically increasing within-pool combining abilities and (2) to identify hybrids with high yield potential and stability. The general combining ability (GCA) of a parent is the average performance of the genotype in all hybrid combinations compared with the mean value of all hybrids involved, whereas specific combining ability (SCA) of a pair of parental lines is defined as the deviation of the realized hybrid performance from the expectation based on GCAs of the parents and the population mean (Sprague and Tatum 1942). Henceforth, GCA is largely due to additive effects and SCA is attributed to the dominance and nonadditive epistatic effects (Falconer and Mackay 1983).

Bernardo (1996) used genetic covariance matrices among tested and untested single crosses to predict best linear unbiased predictions (BLUPs) of untested single crosses from tested single crosses. The genetic value of a large number of untested individuals can be predicted using genetic markers as the cost of genotyping has decreased and computational efficiency has increased. Genomic prediction (GP) uses statistical models to estimate marker effects or model genomic relationships in a training population with phenotypic and genotypic data which is then used to predict the genetic value of individuals solely from genomic information (Meuwissen *et al.* 2001; Bernardo and Yu 2007; VanRaden 2008). Early studies on application of GP for hybrid breeding involved prediction of testcross performance in maize (Albrecht *et al.* 2011; Riedelsheimer *et al.* 2012; Windhausen *et al.* 2012). Results in maize led to studies in several crop species to exploit GP for testcross performance of hybrids (Reif *et al.* 2013; Zhao *et al.* 2013; Jan *et al.* 2016; Philipp *et al.* 2016). More recently, various prediction models and optimization strategies for exploiting genomic relationship as well as nonadditive effects have been reported for hybrid breeding (Guo *et al.* 2019; Varshney *et al.* 2021). GP studies in sorghum have been reported only very recently and the number of studies has been severely limited. Despite the limited exploration, the empirical results from prediction for hybrid sorghum have been promising in the application of genomic selection for sorghum breeding (Hunt *et al.* 2018; Velazco *et al.* 2019; Fonseca *et al.* 2021). One of the key considerations in implementation of GP is the design of the training population (Jannink *et al.* 2010). The degree of relatedness between training and validation sets can affect the accuracy of GP of single-cross hybrids, and therefore, effective design of training population to maximize genomic selection accuracy requires optimization of genomic relationship between training and testing sets (Technow *et al.* 2014; Kadam *et al.* 2016; Labroo, Ali, *et al.* 2021). The studies on the effect of genomic relationship on prediction accuracy of hybrids have not yet been reported in sorghum, and empirical studies examining such hypotheses are crucial for effective implementation of GP for hybrid prediction in sorghum.

In this study, we utilized the extant genetic diversity of grain sorghum to design and characterize a diverse panel of single-cross hybrids created using a large number (301) of R lines crossed to two female testers. The male lines used for generating single crosses are accessions included within the US sorghum association panel (SAP) that represent the genetic and phenotypic diversity of sorghum across the globe and could be used in allele mining and prebreeding (Casa *et al.* 2008). Our objectives were (1) to characterize heterotic performance for testcross hybrids, (2) to identify diverse male lines with superior combining ability for incorporation into the pollinator parent gene pool, and (3) to compute accuracies of GP of hybrid genetic value and male GCA. We used high density genomic markers in the framework of genomic best linear unbiased prediction (GBLUP) models for GP of hybrid and line performance.

## Materials and methods

### Plant materials and population design

A sorghum hybrid diversity panel (SHDP) of 602 hybrids was generated by crossing 301 diverse male restorer (R) lines to two CMS female (A) testers: ATx642 (PI656029) and ATx2928 (PI629059) (Supplementary File 1, Supplementary Fig. 1). The male and female lines were selected to maximize diversity and phenotypic performance. ATx642 is durra type female tester known for its post-flowering drought tolerance, and ATx2928, a kafir type, is a female tester with white pericarp commonly used for development of food grade hybrids. The male lines and female testers used for single-cross development are accessions included within the US SAP, and therefore, represent the genetic and phenotypic diversity of global sorghum germplasm (Casa *et al.* 2008). Parents were grown in Bahia de Banderas, Nayarit, Mexico (approximately 20.78473 N, 105.24865 W) in the winter nursery of years 2018 and 2019 to make single-cross hybrids in tester × line (A × R) factorial design. Therefore, the hybrids in the SHDP can be grouped into two half-sib families based on the common female tester. The parental lines were used to estimate MPH, and a check hybrid (83P17; Pioneer) recommended for South Carolina was used to estimate commercial relative performance (Labroo, Ali, *et al.* 2021) of the experimental single cross hybrids.

### Field design and phenotyping

The hybrids and parental lines from the SHDP were planted along with the commercial check in a modified randomized complete block design with two replications in May 2019 and May 2020 at the Clemson University Pee Dee Research and Education Center (2019: 34.29506 N, 79.74605 W; 2020: 34.29058 N, 79.74362 W) in Florence, SC. The modification was done by blocking each complete replication into smaller blocks based on previously observed maturity and height phenotypes for the male parent of the hybrids to avoid shading effect of taller lines on neighboring plants. There were a total of 40 blocks per replication and each block consisted of 30 individual plots. Each plot had two rows with a row length of 6.096 m and a row spacing of 0.762 m. The experimental hybrids, their parental lines and check hybrid variety (83P17) were randomized completely for each small block nested within each replication. Preplant fertilizer was blended based on soil sample results and per field recommendations for cereal grain production. In 2019, the fertilizer blend consisted of granular muriate of potash (KCl; 0-0-60) at 300 kg ha^−1^ as well as monoammonium phosphate (11-52-0) at 125 kg ha^−1^. In 2020, application rates for potash and monoammonium phosphate were 315 and 110 kg ha^−1^, respectively. Seed for all sorghum entries were treated with a blend of fluxofenim (Concep, herbicide antidote), clothianidin (Nipsit, insecticide), mefenoxam (Apron XL, fungicide), and fludioxonil (Maxim XL, fungicide). Immediately after planting, fields were sprayed with a pre-emergent herbicide containing atrazine and S-metolachlor to prevent germination of weeds. At approximately 40 days after planting each season, urea ammonium nitrate was side-dressed at 90 kg ha^−1^. Sugarcane aphids (Mela) were controlled with one or more applications of flupyradifurone (Sivanto Prime), and chlorantraniliprole (Prevathon) was administered in a single application to prevent corn earworm (*Helicoverpa zea*) and fall armyworm (*Spodoptera frugiperda*) infestation. Fields were watered using overhead irrigation only as needed to prevent drought stress.

Phenotypic data were collected for days to anthesis (DTA) when about 50% of the primary panicles in the plot were at mid-anthesis. Plant height (PH) was measured from the base of the plant to the top of the representative panicle within the plot after physiological maturity. Plants were harvested when the majority of the plants in the plot reached post-physiological maturity (grain moisture below 18%) using a Wintersteiger Delta two row combine plot harvester. Harvest dates ranged from 16 to 18 September in 2019 and 17 to 25 September in 2020. Total harvest weight of the grain from each plot and the average moisture content of the harvested grain was used to calculate grain yield (GY) in bushels/acre and subsequently converted to ton per hectare (t ha^−1^). Plots with harvest weight less than 1,000 g because of poor plant stand were set as missing to avoid the outlier effect, and to avoid any confounding due to machine error in moisture reading we imputed the plots with moisture content below 10% with the mean moisture content from the same year for calculation of grain yield per area. A random sample of harvested grain was used to measure 1,000 grain weight (TGW) using Discovery series scale (Ohaus, Parsippany, NJ) and grain number per plot (GNP) was estimated using harvested grain weight and TGW for each plot.

In order to evaluate the heterotic potential of each hybrid combination, we adjusted phenotypic means of parental lines and hybrids for replication and block effect within each year. Isogenic maintainer (B) lines of the female parents were used to evaluate female parent performance. The following model was used to adjust for spatial variability within each experimental year:


(1) $$y_{ijk}=\mu +g_{i}+r_{j}+b_{(k)j}+\epsilon_{ijk}$$

where$y_{ijkl}$ was the phenotypic response of the ith genotype in the kth block nested in the jth replication, μ was the overall mean, $g_{i}$ was the random effect of the ith genotype with $N(0,I\sigma_{g}^{2})$, $r_{j}$ was the fixed effect of the jth replication, $b_{(k)j}$ was the random effect of the kth block nested in the jth replication with $N(0,I\sigma_{b}^{2})$, and $\epsilon_{ijk}$ was the random error associated with each replicate with $N(0,I\sigma_{e}^{2})$.

The adjusted means (BLUPs) of $F_{1}$ hybrid and the parents were used to calculate MPH for a given hybrid for the experimental year using the following formula:


(2) $$\mathrm{MPH}(\text{\%})=100\;*\;\frac{F_{1}-MPV}{MPV}$$

where$F_{1}$ was the adjusted phenotypic value of the hybrid and MPV was the adjusted parental phenotypic value for each year. We also evaluated the IMPH to assess population level heterosis. IMPH is defined as the difference between the mean of the $F_{1}$ and the mean of the inbred parent populations as described in Lamkey and Edwards (1999) shown by the formula below:


(3) $$\mathrm{IMPH}=\hat{H}-\hat{P}$$

where$\hat{H}$ and $\hat{P}$ are the mean of the experimental hybrid population and inbred parent population, respectively.

### Molecular marker data

Genome-wide markers for the inbred parents were generated from whole genome resequencing using Illumina NovaSeq 6000. The sequenced reads were aligned to version 3.1.1 of sorghum reference genome BTx623 (McCormick *et al.* 2018), single nucleotide polymorphism variants were called using joint calling in GATK pipeline and imputed using Beagle at ∼99% accuracy [for details see Boatwright *et al.* (2021)]. The phased genotypic data were filtered using *vcftools* (Danecek *et al.* 2011) to only allow 1% missingness and 2% minor allele frequency resulting in 7,804,754 total SNPs, which was further thinned using *vcftools* to only include one SNP marker every 100 bp to a total SNP count of 2,687,342 for computational efficiency. Hybrid marker genotypes were imputed using *build.HMM* function in R package sommer (Covarrubias-Pazaran 2016) from the parental marker genotype. Additive relationship matrices were calculated using the function *A.mat* (Endelman and Jannink 2012) in sommer separately for inbred genotypes and hybrid genotypes.

### Modeling and statistical analysis

All linear mixed models for variance component analysis and GPs were fit using the single step linear mixed model in sommer (Covarrubias-Pazaran 2016). Phenotypic values of each genotype from small block within the replication nested within year were considered unit of observation in the study.

#### Phenotypic analysis

Genetic variances for DTA, PH, GY, TGW, and GNP were estimated using the following linear mixed model:


(4) $$Y_{ijkl}=\mu +H_{i}+E_{j}+HE_{ij}+B_{((k)l)j}+\epsilon_{ijkl}$$

where$Y_{ijkl}$ was the phenotypic response of the ith single-cross hybrid genotype in the kth block nested in the jth year and lth replicate, μ was the overall mean, $H_{i}$ was the random effect of the ith hybrid genotype with $N(0,I\sigma_{H}^{2})$, $E_{j}$ was the fixed effect of the jth year, $HE_{ij}$ was the random interaction effect of the ith hybrid and the jth year with $N(0,I\sigma_{HE}^{2})$, $B_{((k)l)j}$ was the random effect of the kth block nested in the lth replication within the jth year with $N(0,I\sigma_{B}^{2})$, and $\epsilon_{ijkl}$ was the random error associated with each replicate with $N(0,I\sigma_{e}^{2})$.

The broad sense heritability was estimated for each trait from the variance components in model (4) using the following formula:


(5) $$H^{2}=\frac{\sigma_{H}^{2}}{\sigma_{H}^{2}+\sigma_{HE}^{2}/n_{E}+\sigma_{e}^{2}/n_{E}\;*\;n_{R}}$$

where$\sigma_{H}^{2}$ was the variance among hybrid genotypes from model (4), $\sigma_{HE}^{2}$ was the variance of interaction term between hybrid genotype H and year E, $\sigma_{e}^{2}$ was the error variance, and $n_{E}$ and $n_{R}$ were the total number of year and replication in the study, respectively.

Additive genetic variance of each trait was estimated from the model equivalent to model (4) with the additive genomic relationship matrix of hybrids replacing the identity matrix. The random effect H was assumed to have multivariate normal (MVN) distribution with $H∼\mathrm{MVN}(0,G_{H}\sigma_{H_{a}}^{2})$, where $G_{H}$ was the additive genomic relationship matrix of the $F_{1}$ hybrids and $\sigma_{H_{a}}^{2}$ is the additive genetic variance of the $F_{1}$ hybrid, and the random effect of interaction between hybrid and year was fit with variance–covariance matrix derived from Kronecker product of additive relatedness matrix ($G_{H}$) and homogeneous diagonal matrix for year, E (Bernardo 2002). Narrow-sense heritability ($h^{2}$) was computed from the additive genetic variance using the following formula:


(6) $$h^{2}=\frac{\sigma_{H_{a}}^{2}}{\sigma_{H}^{2}+\sigma_{HE}^{2}+\sigma_{e}^{2}}$$

where$\sigma_{H_{a}}^{2}$ was the additive variance among hybrid genotypes, $\sigma_{H}^{2}$ was the total genetic variance of hybrid genotypes from model (4), $\sigma_{HE}^{2}$ was the variance of interaction term between hybrid genotype H and year E, and $\sigma_{e}^{2}$ was the error variance.

#### Genomic prediction

GBLUP of hybrid genetic value and male GCA were estimated for each trait using two separate models: the genomic GCA model and the genomic GCA+SCA model. Since we only had two female testers in the study, sample size was inadequate for the prediction of GCA of females, therefore we fit testers as fixed effect rather than random effect in our model. Model fit between the two models was evaluated based on the AIC and BIC of models for each trait. The genomic GCA model was


(7) $$Y_{ijklm}=\mu +F_{i}+M_{j}+ME_{\;jk}+E_{k}+B_{((l)m)k}+\epsilon_{ijklm}$$

where$Y_{ijklm}$ was the random phenotypic response of a single-cross hybrid of the ith female line and the jth male line observed in the kth year, lth block, and mth replicate, μ was the overall mean, $F_{i}$ was the fixed effect of the ith female tester, $M_{j}$ was the random effect of the jth male parent with $M∼\mathrm{MVN}(0,G_{M}\sigma_{M}^{2})$ where $G_{M}$ was the additive genomic relationship matrix for males lines, $E_{k}$ was the fixed effect of the kth year, $B_{(l)k}$ was the random effect of the lth block nested in the mth replication within the kth year with $N(0,I\sigma_{B}^{2})$, $ME_{\;jk}$ was the random interaction of the jth male and kth year with $N(0,EG_{M}\sigma_{ME}^{2})$ where $EG_{M}$ was the Kronecker product of the identity matrix (E) for year and $G_{M}$ (Bernardo 2002), and $\epsilon_{ijklm}$ was the random error of each observation with $N(0,I\sigma_{e}^{2})$.

The genomic GCA+SCA model was


(8) $$Y_{ijklm}=\mu +F_{i}+M_{j}+ME_{\;jk}+FM_{ij}+FME_{ijk}+E_{k}+B_{((l)m)k}+\epsilon_{ijklm}$$

wheremodel terms were as described above in (7), $FM_{ij}$ was the random SCA interaction effect of the ith female and the jth male parental lines, with $FM∼\mathrm{MVN}(0,G_{FM}\sigma_{FM}^{2})$, $G_{FM}$ was the Kronecker product of $G_{F}$ and $G_{M}$ (Bernardo 2002), and $FME_{ijk}$ was the random interaction of the ith female, jth male, and kth environment with $N(0,EG_{FM}\sigma_{FME}^{2})$ where $EG_{FM}$ was the Kronecker product of identity matrix for year and $G_{FM}$.

Hybrid genetic value for single-cross hybrids was computed for the GCA model using the following formula:


(9) $$\hat{y_{ij}}=\hat{\mu }+\hat{\;f_{i}}+\hat{m_{j}}$$

andfor GCA+SCA model using the following formula:


(10) $$\hat{y_{ij}}=\hat{\mu }+\hat{\;f_{i}}+\hat{m_{j}}+\hat{s_{ij}}$$

where$\hat{y_{ij}}$ was the hybrid genetic value, $\hat{\mu }$ was the overall mean, $\hat{\;f_{i}}$ was the fixed effect of the ith female tester, $\hat{m_{j}}$ was the GCA of the jth male line, and $\hat{s_{ij}}$ was the SCA of ith female tester and jth male line.

BLUPs of hybrid genetic value and male GCAs were also estimated using the model (7) and (8) by replacing genomic relationship matrices with identity matrices of equal rank. The square root of reliability of GP was calculated as Pearson’s correlation between GBLUP of hybrid genetic value (or male GCA) and BLUP of hybrid genetic value (or male GCA) when all observations were used for estimation of both.

All pairwise comparison of mean between groups was done using Student’s t-test in R software (Team *et al.* 2019). The genetic distances between the two parents were estimated using the above-mentioned thinned marker data and *dist( )* function in R (Team *et al.* 2019).

### Evaluation of prediction accuracy

**Fig. 1.**
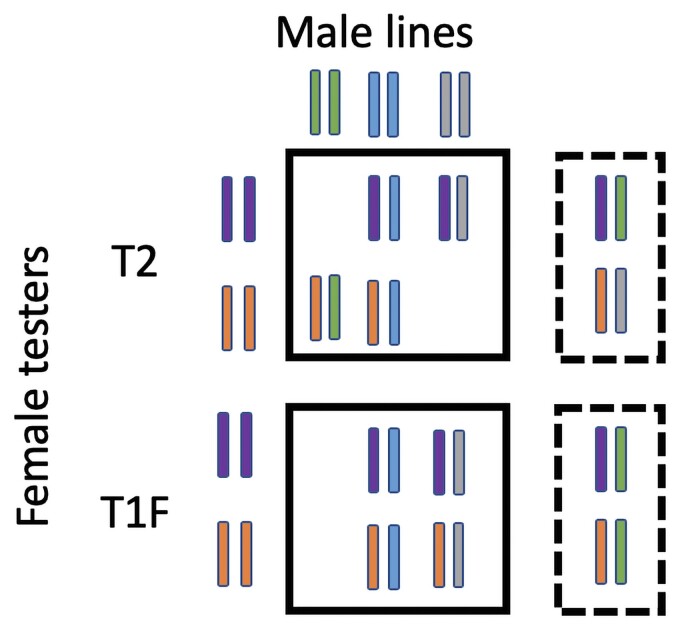
Schematic visualization showing distribution of hybrids into training and testing sets for evaluation of hybrid performance in T2 and T1F hybrids. Solid rectangles represent training sets and rectangle with broken lines represent validation sets.

The evaluation of prediction accuracy of models was done by sampling 80% of the hybrids into training set and remaining 20% were included in the validation set. For prediction of hybrid genetic values, two distinct types of training and validation methods were conducted to test the effect of varying genetic relatedness between hybrids in training and testing sets as previously described in Technow *et al.* (2014). As shown in Fig. 1, both male lines and female testers of the hybrids in T2 validation sets were represented in the training set, whereas only the female parents for hybrids in T1F validation sets were included in the training set. For the prediction of male GCA, we randomly sampled 60 male lines and masked the phenotypes of the hybrids from those male parents in the training set and those 60 male lines were considered as the validation set. A total of 100 iterative sampling was done and R function *set.seed* was used to set the seeds from 123 to 222 for sampling. The predictive ability was calculated as Pearson’s correlation coefficient (r) between predicted values and observed phenotypic values (BLUPs) for the individuals in testing set. Prediction accuracy was then measured as predictive ability divided by square root of reliability of prediction, which was calculated as correlation between BLUPs and GBLUPs calculated for the complete set of hybrids.

## Results

### Trait summary and heterosis

**Fig. 2.**
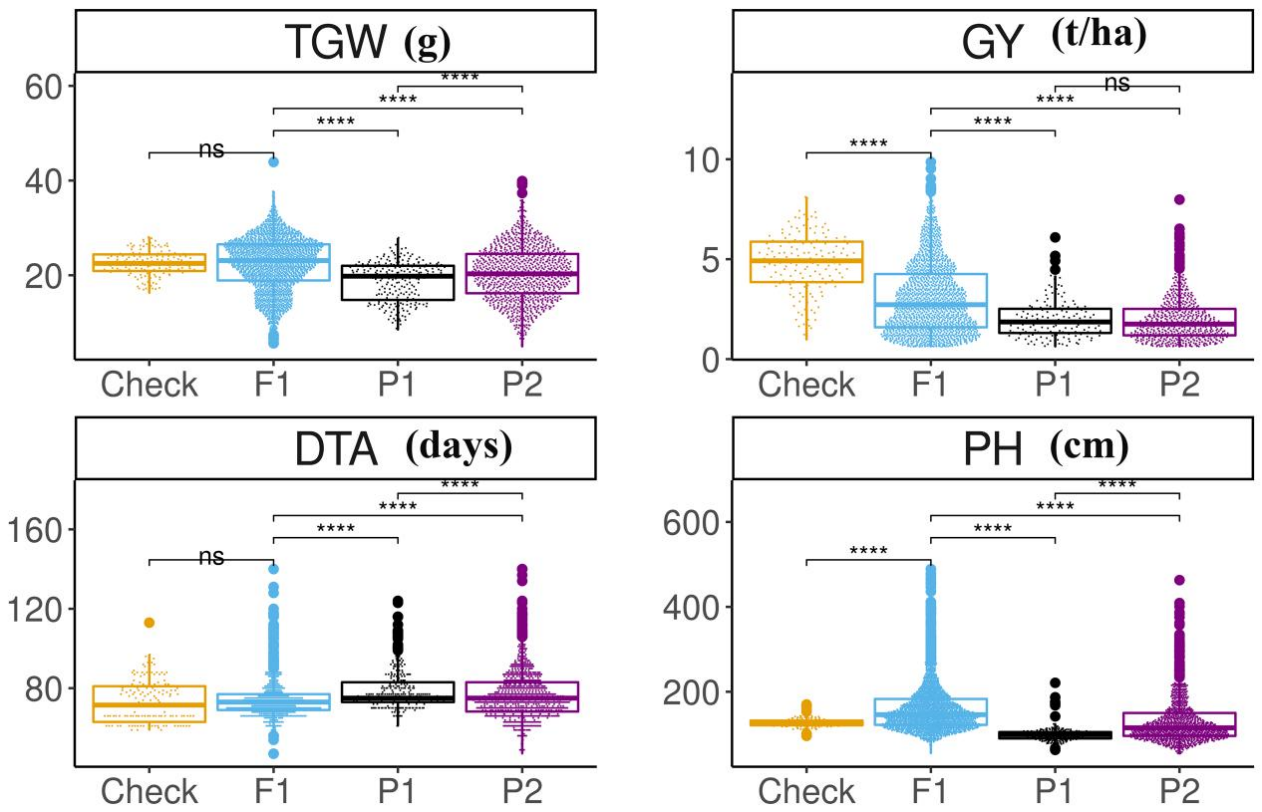
Comparison of agronomic trait performance between the $F_{1}$ hybrids, their parents, and check hybrid. DTA, days to anthesis; GY, grain yield; PH, plant height; and TGW, thousand grain weight. P1 represents female parents and P2 represents male parents. Pairwise comparison done using t-test; ****, $P<1\times 10^{-10}$, and ns, not significant.

The experimental $F_{1}$ hybrids had significantly higher mean than the parental lines for PH, TGW, and GY, whereas the mean values were significantly lower in hybrids than the parental lines for DTA (Fig. 2). The check hybrid showed significantly higher GY and lower PH than the $F_{1}$ hybrids, but there was no significant difference between the two for DTA and TGW (Fig. 2). The phenotypic values between years were significantly different ($P<1\times 10^{-6}$) for each trait (Supplementary Fig. 2). The overall mean phenotypic values for the hybrids were 74 days, 163 cm, 2.9 t ha^−1^, 22.4 g, and 97,587 seeds for DTA, PH, GY, TGW, and GNP, respectively (Table 1). The mean phenotypic values of all traits except PH were significantly different between the half-sib family of hybrids that shared the common female parent. The phenotypic mean was higher for ATx642 hybrids compared to ATx2928 for all traits except PH and TGW (Table 1).

**Table 1.** Mean and standard deviation (in parentheses) for phenotypic values and mid-parent heterosis.

Trait	Phenotypic values			Mid-parent heterosis (%)		
	Whole	ATx2928	ATx642^a^	Whole	ATx2928	ATx642^a^
DTA (d)	74 (9)	71 (8)	76 (9)***	− 4.0 (5.7)	− 3.5 (5.7)	− 4.4 (5.6)**
GNP	97,587 (53,485)	87,662 (51,945)	106,662 (53,292)***	28.2 (39.0)	18.2 (31.5)	38.5 (43.2)***
GY (t ha^−1^)	2.9 (1.7)	2.7 (1.6)	3.2 (1.8)***	39.3 (48.4)	32.4 (42.5)	46.2 (52.9)***
PH (cm)	163 (60)	162 (58)	163 (62)	38.8 (26.4)	38.5 (27.8)	39.0 (24.9)
TGW (g)	22.4 (5.6)	23.3 (5.4)	21.6 (5.6)***	12.7 (16.9)	18.5 (16.9)	6.9 (14.6)***

^*a*^Mean comparison was calculated between hybrids from the two female parents. *, **, and *** correspond to P-values < 0.05, 0.01, and 0.001, respectively. DTA, days to anthesis; PH, plant height; GNP, grain number per plot; GY, grain yield; and TGW, thousand grain weight.

**Fig. 3.**
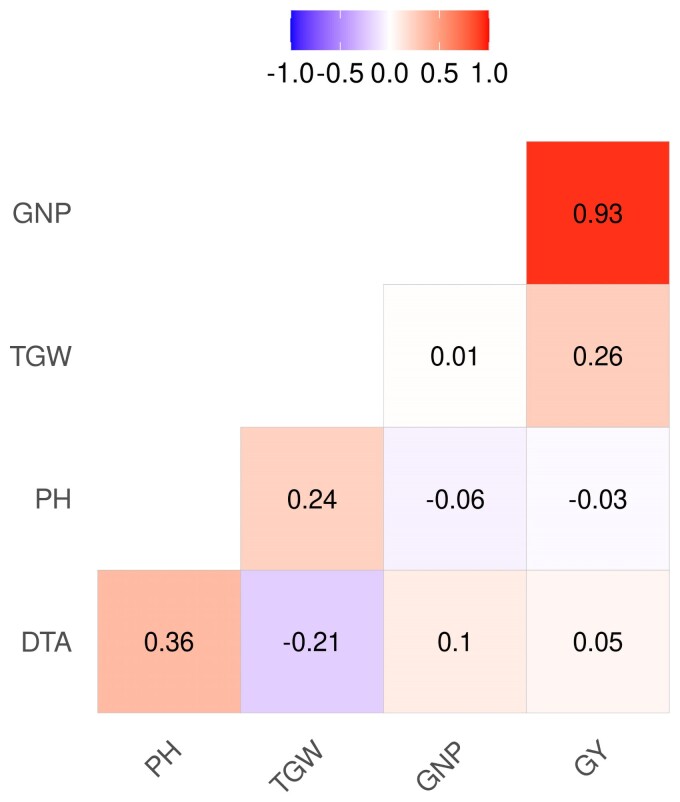
Correlation between adjusted phenotypic values of agronomic traits. DTA, days to anthesis; PH, plant height; GNP, grain number per plot; GY, grain yield; and TGW, thousand grain weight.

All traits except DTA exhibited positive MPH (Table 1). Since early flowering (maturity) is the desired outcome, the hybrids exhibited favorable MPH for DTA as well. Overall mean MPH (%) for DTA, PH, GNP, GY, and TGW were −4, 39, 28, 39, and 13, respectively. Mean MPH was significantly higher (P<0.001) for GY and GNP in 2019 than in 2020, whereas there was no significant mean difference (P<0.05) for PH and TGW between the two years (Supplementary Table 1). There was no significant difference in mean MPH for PH between the hybrids based on the female parent pool, but the two families of hybrids were significantly different (P<0.01) for DTA, GNP, GY, and TGW (Table 1). We ran correlations among traits for the $F_{1}$ hybrids using the BLUPs of hybrid genetic value. GY showed a strong positive correlation with GNP and a weak but positive correlation with TGW, whereas TGW and GNP were not correlated to each other (Fig. 3). PH showed a slightly positive correlation to DTA and TGW, while DTA and TGW were slightly negatively correlated. The traits were also positively correlated across the years with correlation ranging from 0.37 for GNP to 0.82 for PH (Supplementary Fig. 3).

The commercial check hybrid used in our study was likely to be bred and recommended based on a selection index that is unavailable to us, so the true comparison of our experimental hybrids to commercial check based on a multitrait index was out of scope. On average, our experimental hybrids had lower mean hybrid performance than the commercial check except for TGW in 2019, DTA in 2020, and PH for both years. Among our experimental hybrids, a total of 27 hybrids in 2019 and 3 hybrids in 2020 had higher grain yield than the commercial check, with the best hybrid in 2019 yielding 41% over the commercial check. Since MPH and commercial heterosis exhibited a strong positive correlation, the hybrids that had high MPH were more likely to also perform well compared to commercial check hybrid (Supplementary Fig. 4). MPH and genetic distance between parents were significantly positively correlated for PH with slightly better correlation (0.28) for ATx642 hybrids than ATx2928 hybrids (0.16), whereas no significant correlation was observed between MPH and parental genetic distance for grain yield components except for TGW (−0.11) in ATx2928 hybrids (Supplementary Table 2). The estimates of IMPH showed that on population level our experimental hybrids were taller, had earlier flowering, greater seed number and seed size, and had higher grain yield than the parental population (Supplementary Table 3).

### Variance components and heritabilities

Variance component analysis from model (4) showed that variances due to hybrid genotype, genotype × year, and blocks within replication within year had significant contribution to total phenotypic variance (Supplementary Table 4). Environmental effects were largely from the blocks within replication and variance due to year was mostly insignificant, so we fit year as a fixed effect in dependent models. PH had the highest broad and narrow-sense heritabilities, whereas TGW had the lowest broad and narrow-sense heritabilities (Table 2). Broad sense heritabilities ranged from 0.63 to 0.92 and narrow-sense heritabilities ranged from 0.44 to 0.86 (Table 2). Broad sense heritabilities of GY were 0.57 and 0.69 for hybrids from female parents ATx2928 and ATx642, respectively.

**Table 2.** Heritability estimates for agronomic traits in the single-cross hybrids.

Trait	$H^{2}$	$h^{2}$
DTA	0.79 (0.02)	0.77 (0.02)
PH	0.92 (0.01)	0.86 (0.01)
GNP	0.67 (0.04)	0.48 (0.05)
GY	0.64 (0.04)	0.49 (0.05)
TGW	0.63 (0.03)	0.44 (0.05)

Values in parentheses show standard error of respective heritability estimate. DTA, days to anthesis; $H^{2}$, broad sense heritability; $h^{2}$, narrow-sense heritability; GNP, grain number per plot; GY, grain yield; PH, plant height; and TGW, thousand grain weight.

Due to the limitation in the number of females testers used to generate single-cross hybrids, we assumed female testers to have fixed effects in models (7) and (8). Supplementary Table 5 shows the fixed effect estimates for overall mean, year, and female tester effect for GCA and GCA+SCA models. However, SCA effects in GCA+SCA model were estimated by fitting of random interaction effect between the female tester and male line. Table 3 shows proportion of variance for male GCA and single-cross SCA in models (7) and (8) for hybrid phenotypic values of all five traits. Variance for phenotypic values among single crosses ($\sigma_{\mathrm{mGCA}}^{2}$ and $\sigma_{\mathrm{SCA}}^{2}$) was significantly different from zero in all cases except $\sigma_{\mathrm{SCA}}^{2}$ for grain yield (α = 0.05). The proportion of $\sigma_{\mathrm{SCA}}^{2}$ was the greatest for PH followed by GNP (Table 3).

**Table 3.** Proportion of variance due to male GCA ($\sigma_{\mathrm{mGCA}}^{2}$) and single-cross specific combining ability ($\sigma_{\mathrm{SCA}}^{2}$) for hybrid values.

Model	Trait	$\sigma_{mGCA}^{2}$	$\sigma_{SCA}^{2}$
GCA (7)	DTA	0.37***	—
	PH	0.71***	—
	TGW	0.34***	—
	GY	0.24***	—
	GNP	0.23***	—
GCA+SCA (8)	DTA	0.37***	0.04**
	PH	0.63***	0.15***
	TGW	0.31***	0.05**
	GY	0.22***	0.04
	GNP	0.20***	0.06*

*, **, and *** correspond to P-values <0.05, 0.01, and 0.001, respectively. DTA, days to anthesis; GNP, grain number per plot; Gy, grain yield; PH, plant height; and TGW; thousand grain weight.

### Prediction of hybrid performance in T2 and T1F scenarios

For genomic GCA and genomic GCA+SCA models, we evaluated prediction accuracy for T2 and T1F scenario for the hybrid phenotypic values. Prediction accuracies were always significantly greater when training and validation sets shared both parents (T2) compared to when only the female parents (T1F) were shared (Table 4). For the genomic GCA model, the average prediction accuracies for T2 scenario ranged from 0.87 (GNP) to 0.93 (PH), and the average prediction accuracies for T1F scenario ranged from 0.09 (PH) to 0.48 (GNP) (Table 4). Mean prediction accuracies were always greater with genomic GCA model compared to genomic GCA+SCA model for T2 prediction scenario but there was no significant difference between the two models within the T1F prediction scenario with the exception of TGW. Decline in prediction accuracy from T2 to T1F scenario ranged from 40% (GNP) to 90% (PH).

**Table 4.** Mean and standard deviation (in parentheses) of prediction accuracies for hybrid performance for hybrid values across prediction scenarios and models used.

Trait	T2		T1F	
	GCA	GCA+SCA	GCA	GCA+SCA
DTA	0.91 (0.04)	0.85 (0.05)	0.29 (0.12)	0.28 (0.12)
GNP	0.87 (0.03)	0.76 (0.05)	0.48 (0.08)	0.45 (0.08)
GY	0.88 (0.03)	0.80 (0.04)	0.38 (0.09)	0.36 (0.10)
PH	0.93 (0.02)	0.76 (0.04)	0.09 (0.11)	0.09 (0.10)
TGW	0.91 (0.02)	0.81 (0.03)	0.13 (0.08)	0.13 (0.08)

DTA, days to anthesis; GNP, grain number per plot; GY, grain yield; PH, plant height; and TGW, thousand grain weight.

### Prediction of line performance

**Fig. 4.**
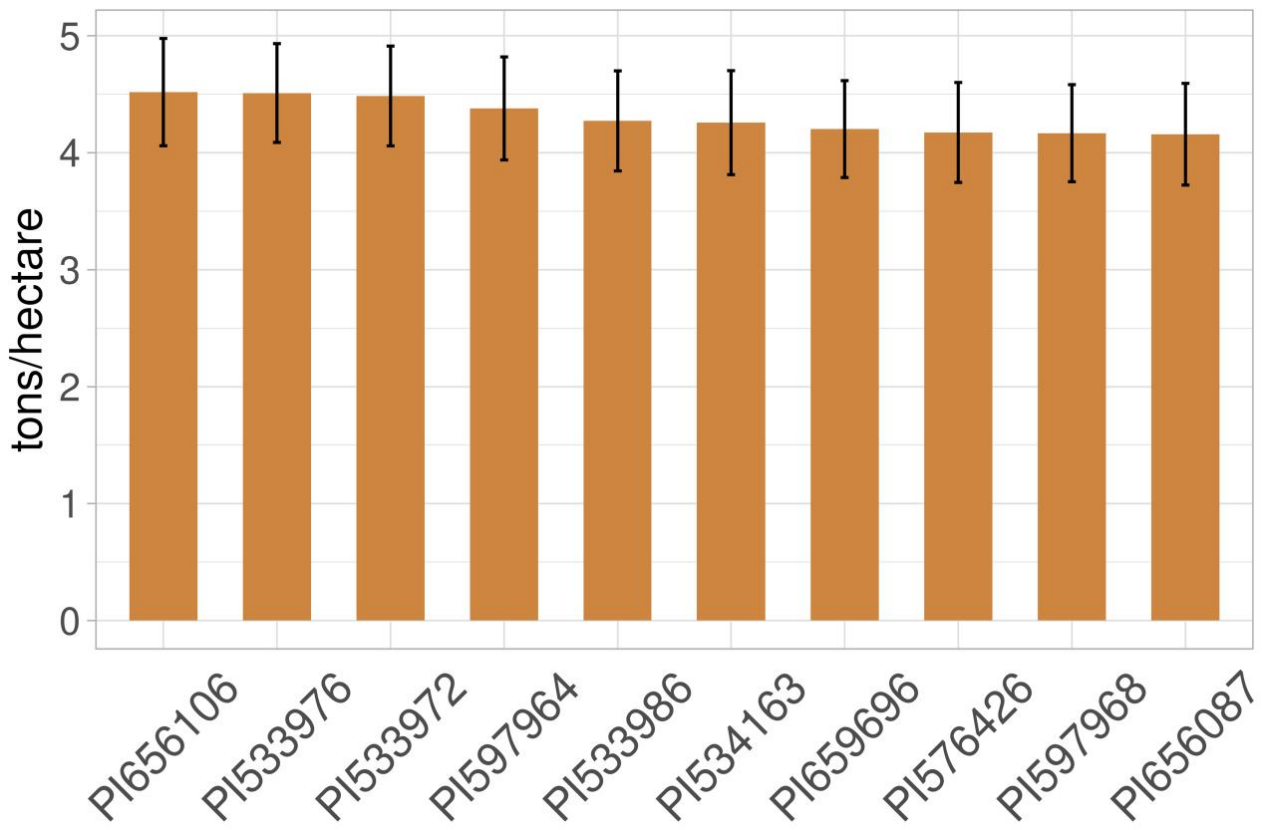
Male lines with the highest GCA for grain yield from the genomic GCA model (7) when all observations were used. Error bars represent standard error for the GBLUPs of GCA.

GCAs of male lines were estimated from the genomic GCA model (7) using all observations in the computation. Figure 4 shows top 10 male lines with the highest GBLUPs for GCA all observations for grain yield were used as response in the GCA model (7).

**Fig. 5.**
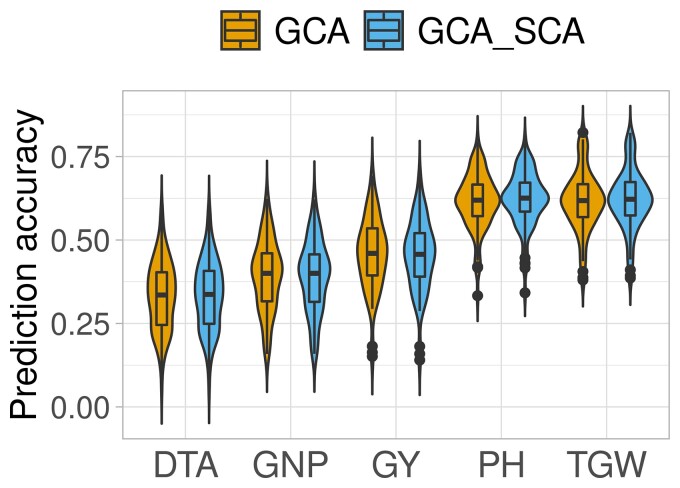
Prediction accuracy of male GCA for hybrid phenotypic values. DTA, days to anthesis; PH, plant height; GNP, grain number per plot; GY, grain yield; SCA, specific combining ability; and TGW, thousand grain weight.

The ability to predict testcross performance in hybrids was evaluated for male general combining abilities (mGCA) using the GCA and GCA+SCA model for hybrid values. Model parameters (AIC and BIC) used to evaluate the two models (GCA and GCA+SCA) showed GCA+SCA model to perform slightly better, but no significant differences were observed for mean prediction accuracies between the two models (Supplementary Table 6, Fig. 5). Mean prediction accuracies of male GCA for hybrid performance were 0.33, 0.62, 0.39, 0.62, and 0.46 for DTA, PH, GNP, TGW, and GY, respectively (Fig. 5).

## Discussion

Hybrid sorghum breeding is based on a CMS system where male sterile females (A lines or seed parents) are crossed to restorer males (R lines or pollinator parents) to produce seed of commercial $F_{1}$ hybrids. Therefore, the CMS system imposes heterotic groups based on fertility reaction of sorghum genotypes and not solely on genetics (Crozier *et al.* 2020). Consistent heterotic advantage is observed in sorghum hybrids from crossing A × R lines which most likely arises from baseline heterosis due to outbreeding rather than from separation of pools. Unlike in maize, there is often no clear separation of genetics between the A and R line pools in sorghum. The genetic structure resulting from evolutionary divergence and subsequent population subdivision within the foundational sorghum accessions can lead to lack of marked genetic distance between heterotic groups (Brown *et al.* 2011; Crozier *et al.* 2020). One of the important goals in breeding sorghum hybrids is to identify pollinator parents that have good combining ability and are capable of restoring the fertility of A lines in the hybrid combination. In this study, heterotic potential and combining abilities were examined for a large number of diverse male restorer lines crossed to two established female testers. The goal was to identify best combiners and evaluate accuracy for GP of hybrid genetic value and GCA of male lines.

### Consistent heterotic advantage for agronomic traits

Following the wide range of diversity in the parental genotypes, the $F_{1}$ hybrids in this study showed tremendous variance in agronomic performance. Mean MPH observed in our population were consistent with previous observations of 30–50% for grain yield and −1% to −6% for days to anthesis, but slightly higher MPH for plant height (39%) than previously reported (up to 20%) (Kirby and Atkins 1968; Jordan *et al.* 2003; Reddy *et al.* 2007; Crozier *et al.* 2020). Trait heritabilities for DTA, PH, and GY observed in our study were moderate to high and were consistent with previous reports in sorghum hybrids (Velazco *et al.* 2019; Fonseca *et al.* 2021). Since the hybrids and the parents were placed within the same block within the replication in our field design, we acknowledge that it could lead to some confounding of spatial variability with genetic effects even after correcting for block and replication effects. Therefore, in addition to measuring heterosis on a single-cross level using MPH, we also estimated population wide heterosis in the form of IMPH as described by Lamkey and Edwards (1999). The IMPH in our population was consistently observed for all the traits but was lower than the MPH for each trait and year combination (Supplementary Tables 1 and 3). The commercial check hybrid outperformed most of our experimental hybrids which is not surprising considering the check variety is bred from elite parents whereas our parental lines are more diverse but less elite, however, there were some hybrid combinations that yielded better than the commercial check.

### Prediction of single-cross performance in T2 and T1F

Since the possible hybrid combinations even in a medium sized breeding program far exceeds the capacity to test them in actual hybrid crosses, one of the main motivation for the use of GP in hybrid breeding is to be able to estimate hybrid genetic value of those untested hybrid crosses (Hallauer *et al.* 2010; Technow *et al.* 2014). Studies in maize have shown that GP can be used to predict hybrid genetic value of single crosses from inbreds in heterotic groups early in the breeding cycle to make selections for elite hybrid development (Technow *et al.* 2014; Kadam *et al.* 2016).

We evaluated accuracies when both parents (T2) or only female parent (T1F) were shared between training and validation sets to understand the effect of parent testing on the accuracy of single cross predictions. While single-cross performance was predicted with high accuracy in T2 scenario, a strong decline (12–90%) in prediction accuracy was observed in T1F compared to T2. This is expected because the predictions from the model in T2 rely not only on kinship between male parents (which is usually low since they come from a diverse panel), as in the case of T1F, but also on information from other crosses in the training set that involves the male parent of predicted hybrid with the other female tester. Plant height showed the greatest decline in prediction accuracy in T1F, which could be due to very limited phenotypic variability among female lines for PH, and as a result, accuracy for PH could have been driven primarily by male parent. GP of sorghum hybrids using GBLUP model and cross-validation involving leave one parent out strategy (similar to our T1F) showed mean prediction accuracies (0.31–0.54) for grain yield that was similar to accuracy from our T1F prediction scenario (Fonseca *et al.* 2021). In rice hybrids, Labroo, Ali, *et al.* (2021) ran a validation strategy similar to this study but did not see as stark a decline in T1F compared to T2 as was observed in this study. However, genotypes in Labroo, Ali, *et al.* (2021) were more closely related and therefore possessed a smaller effective population size, which could have reduced the effective number of loci controlling traits and, as a result, increased the prediction accuracy (Daetwyler *et al.* 2010). Whereas, the SHDP developed for this study was highly structured with varied genetic distance due to inherent population structure and divergence among parental lines. Sapkota *et al.* (2020) have previously reported that racial structure is an important factor that affects GP accuracy in sorghum using inbred lines. Future studies that investigate population structure and its role in hybrid prediction could be useful if GP were to be used in deriving heterotic pools from the subpopulations within sorghum.

### Prediction of male GCA

Prediction of testcross performance of inbred lines in hybrid combinations has been studied across multiple crop species (Albrecht *et al.* 2011; Zhao *et al.* 2013; Jan *et al.* 2016; Hunt *et al.* 2018; Velazco *et al.* 2019). In sorghum, GP of line performance in testcross hybrids for grain yield has reported predictive ability ranging between 0.12 and 0.38 which is consistent with our observation, however, the approach used to calculate of prediction statistics varies between these studies (Hunt *et al.* 2018; Velazco *et al.* 2019). Prediction accuracy for male GCA for grain yield was higher in this study than similar studies in rice (Labroo, Ali, *et al.* 2021) but comparable to that in wheat (Zhao *et al.* 2013). Despite slightly better model fit for genomic GCA+SCA model over genomic GCA model, we did not observe any significant difference between the two models for prediction of male GCA values of any of the traits studied (Supplementary Table 3). Prediction accuracies for heterotic prediction of male GCA were only slightly lower than phenotypic prediction of male GCA but had wider ranges of prediction. Prediction accuracies for prediction of male GCA were higher for PH and TGW compared to DTA, GNP, and GY, which is consistent with previous GP results involving prediction of per se values of these traits using stratified cross-validation in the SAP that included all of the male lines used in this study (Sapkota *et al.* 2020).

### Implication for future of sorghum breeding

The phenotypic distribution of agronomic traits and consistent heterotic advantage of experimental $F_{1}$ hybrids in this study demonstrates the range of genetic diversity in the parental genepool for exploitation of heterotic advantage as well as potential for development of elite hybrids. Furthermore, the application of GP in estimation of hybrid genetic value and GCA can lead to substantial genetic gain by reducing breeding cycle length and increasing response to selection. An important conclusion from our study is that when predicting diverse panel of hybrids, inclusion of both parental lines in at least one hybrid combination in the training set could improve accuracy substantially as opposed to predicting when one of the parents is untested. The conventional wisdom for development of heterotic combinations have relied solely on genetic distance between parents but our results and previous studies show that increased genetic distance does not always translate to increased heterosis or hybrid performance (Falconer and Mackay 1983; Crozier *et al.* 2020; Labroo, Ali, *et al.* 2021).

Studies exploring the role of genetic diversity and genomic relationship on GP for trait phenotypic values as well as trait heterotic values are essential for optimization of training population for predicting hybrid performance. In silico simulation studies have highlighted strategies for application of rapid recurrent genomic selection combined with progeny testing to facilitate separation of heterotic groups, reduction in breeding cycle, and ultimately increase in long term genetic gain (Cros *et al.* 2015; Powell *et al.* 2020; Voss-Fels *et al.* 2021). Estimates of GCA for the diverse set of male lines computed in this study facilitates selection of prospective pollinator parents based on different criteria for exploiting heterosis and hybrid development in sorghum breeding programs. One key consideration for future studies and application of GP in sorghum breeding should be to generate hybrid combinations using diallel or factorial mating design with several diverse group of individuals from each heterotic group to serve as training population to predict hybrid genetic value of untested single crosses rather than focusing on testcross mating scheme (Kadam *et al.* 2016; Fristche-Neto *et al.* 2018; Fonseca *et al.* 2021). We emphasize the need for continuous studies exploring the effect of genetic structure, genomic relationship, and optimization criteria for identifying optimum strategies for genomic selection in sorghum breeding.

## Supplementary Material

jkac311_Supplementary_Data

## Data Availability

The codes and data used for statistical modeling and analysis are available at github.com/sirjansapkota/GP˙Sorghumhybrids. All supplementary tables and figures are available as a single supplementary file. Supplementary File 1 includes information about the parental lines used in hybrid combinations. Supplementary material is available at *G3* online.
