# Supplementary material for: Genomic prediction of hybrid performance for agronomic traits in sorghum
Source: G3 (Bethesda). 2022 Dec 1;13(4):jkac311. doi: 10.1093/g3journal/jkac311 (PMC10085789; doi:10.1093/g3journal/jkac311)
Supplement: jkac311_Supplementary_Data [file jkac311_supplementary_data.pdf]

Genomic prediction of hybrid performance for agronomic traits in sorghum

Sirjan Sapkota[†,*], J. Lucas Boatwright[†,‡], Neeraj Kumar[†], Matthew Myers[†], Alex Cox[†], Arlyn Ackerman[‡,§], William Caughman[§], Zachary E. Brenton[§§], Richard E. Boyles[‡,§] and Stephen Kresovich[†,‡]

Affiliation:
[†]Advanced Plant Technology Program, Clemson University, Clemson, SC, USA
[‡]Department of Plant and Environmental Sciences, Clemson University, Clemson, SC, USA
[§]Pee Dee Research and Education Center, Clemson University, Florence, SC, USA
[§§]Carolina Seeds System, Florence, SC, USA

[*]Corresponding author: ssapkot@clemson.edu

Supplementary information

Figure S1. Principal components (PCs) showing distribution of the male and female parents used the study within the overall diversity panel (SAP). Gray dots represent accessions within the SAP that were not included in this study. The percent variance explained in shown in parantheses and the origin lines are marked by '0' on the top and right side for X and Y axes, respectively.

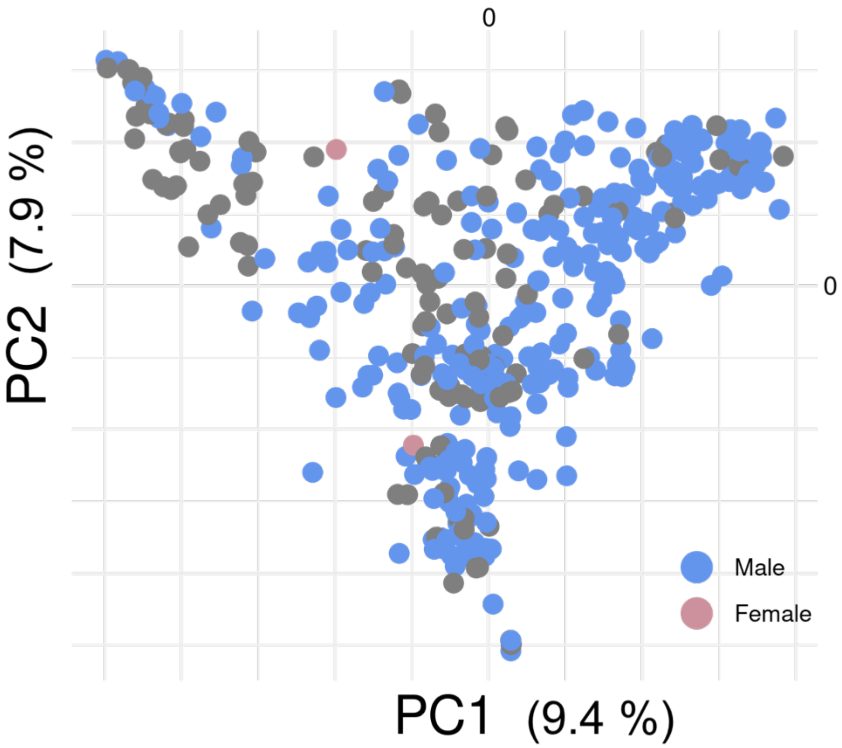

Figure S2. Phenotypic distribution of agronomic and yield traits for the F_1 hybrids.

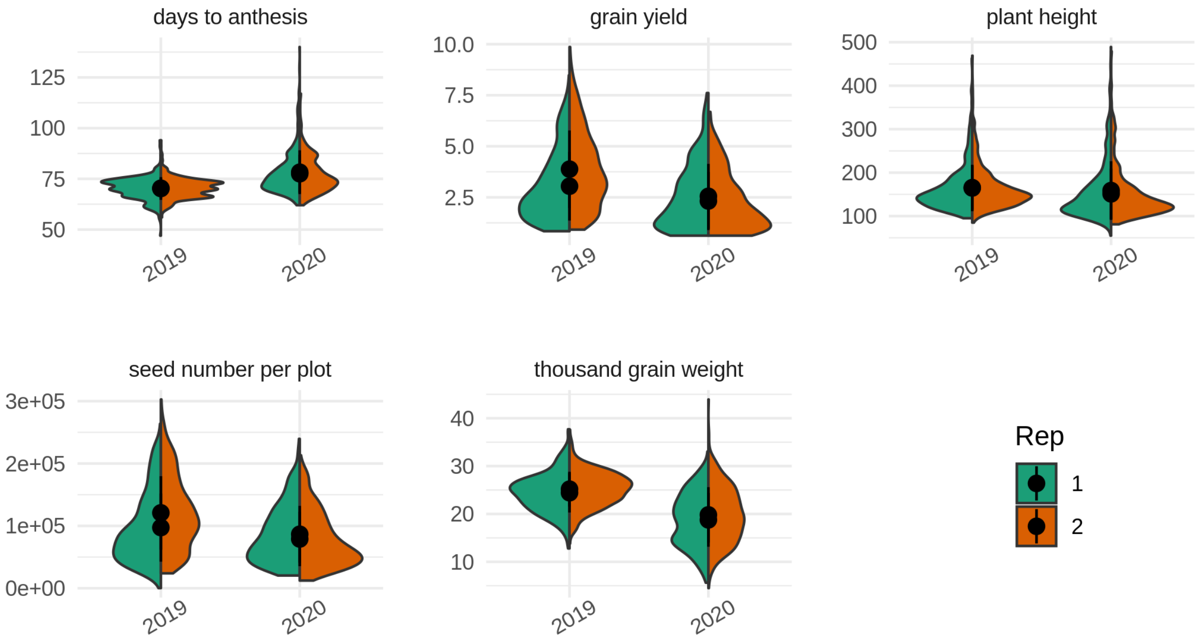

Figure S3. Correlation between phenotypic values of traits across the two field seasons. DTA: days to anthesis, GNP: grain number per plot, GY: grain yield, PH: plant height, and TGW: thousand grain weight.

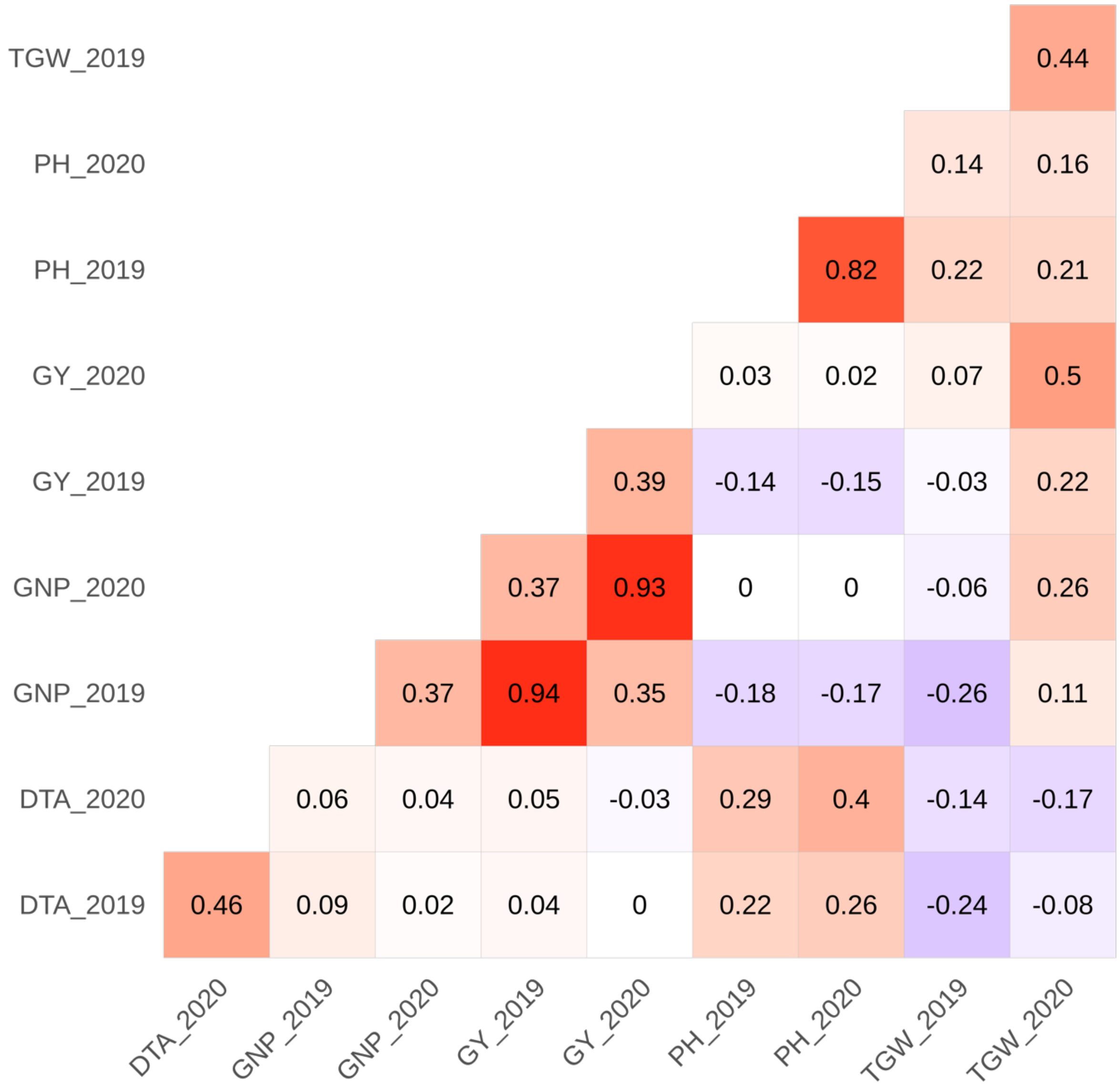

Figure S4. Scatterplot showing distribution of mid-parent heterosis and commercial relative performance (% over check) for F_1 hybrids. DTA: days to anthesis, GNP: grain number per plot, GY: grain yield, PH: plant height, and TGW: thousand grain weight.

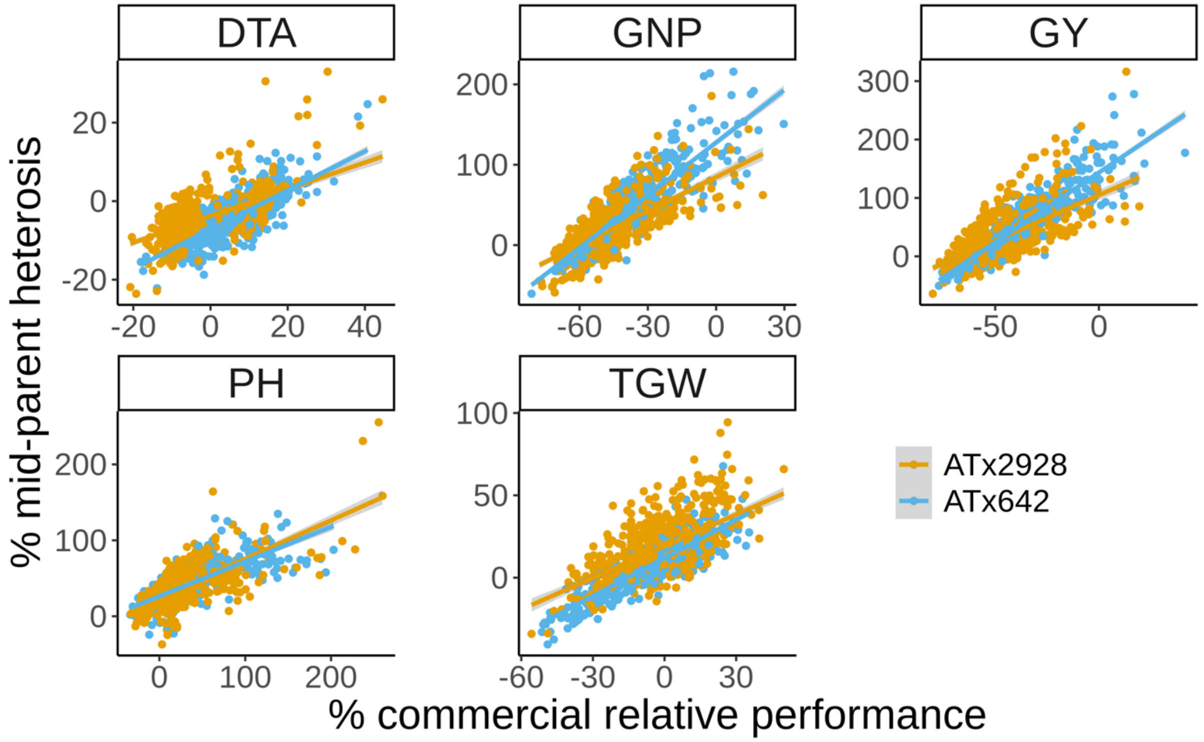

Table S1. Estimates of mid-parent heterosis by experimental year and trait. P-value were calculated from mean comparison between the two experiment years.

	2019	2020	p-value
**DTA**	-1.8 (4.4)	-6.2 (6)	2.20E-16
**GNP**	33.8 (46.2)	22.5 (28.9)	6.50E-07
**GY**	43.8 (50.4)	34.5 (45.8)	0.001
**PH**	38.2 (26.3)	39.4 (26.5)	0.44
**TGW**	12.6 (9.6)	12.9 (22)	7.60E-01

Table S2. Correlation between parental genetic distance and mid-parent heterosis by female parent and traits. DTA: days to anthesis, GNP: grain number per plot, GY: grain yield, PH: plant height, and TGW: thousand grain weight. *** p-value < 0.001.

**Female parent**	**Trait**	**Estimate**	**P-value**
ATx2928	DTA	0.06	0.14
ATx2928	GNP	0.01	0.78
ATx2928	GY	-0.03	0.47
ATx2928	PH	0.16***	8e-05
ATx2928	TGW	-0.11***	8e-03
ATx642	DTA	0.04	0.29
ATx642	GNP	-0.03	0.55
ATx642	GY	-0.01	0.79
ATx642	PH	0.28***	6e-12
ATx642	TGW	0.06	0.17

Table S3. Inbred-midparent heterosis estimates. DTA: days to anthesis, GNP: grain number per plot, GY: grain yield, PH: plant height, and TGW: thousand grain weight.

Trait	Year	F_1 mean	Inbred mean	Inbred-midparent heterosis
DTA	Overall	74.5	77.1	-2.6
	2019	70.4	70.1	0.3
	2020	78.8	82.1	-3.3
GNP	Overall	89197	76117	13080
	2019	100688	84123	16565
	2020	77325	70373	6951
GY	Overall	2.7	2.2	0.5
	2019	3.2	2.6	0.6
	2020	2.2	1.9	0.3
PH	Overall	162	132	30
	2019	167	134	33
	2020	156	131	25
TGW	Overall	21.8	19.7	2.1
	2019	24.7	23.2	1.5
	2020	18.8	17.1	1.7

Table S4. Variance components for linear mixed model for phenotypic analysis. Geno: hybrid genotype, Rep: replication, VarComp: variance component, SE: standard error.

		**Phenotypic values**		
		**VarComp**	**SE**	**Z-ratio**
Days to anthesis	Geno	25.75	1.89	13.65
	Geno x Year	5.69	0.89	6.41
	Year:Rep:Block	18.01	2.23	8.07
	Residual	16.33	0.67	24.25
	Total	65.78		
Plant height	Geno	2470.35	146.47	16.87
	Geno x Year	177.96	25.12	7.09
	Year:Rep:Block	112.43	18.34	6.13
	Residual	448.47	18.24	24.59
	Total	3209.19		
Grain number per plot	Geno	8.53E+08	9.68E+07	8.82
	Geno x Year	2.83E+08	7.94E+07	3.56
	Year:Rep:Block	5.13E+08	7.94E+07	6.46
	Residual	1.14E+09	6.39E+07	17.88
	Total	2.79E+09		
Grain yield	Geno	167.56	20.02	8.37
	Geno x Year	72.51	17.03	4.26
	Year:Rep:Block	116.30	17.54	6.63
	Residual	237.09	12.96	18.30
	Total	593.45		
Thousand grain weight	Geno	7.93	0.82	9.71
	Geno x Year	6.23	0.62	10.04
	Year:Rep:Block	1.85	0.31	5.94
	Residual	6.33	0.29	21.69
	Total	22.34		

Table S5. Fixed effects estimate for GCA and GCA+SCA models for evaluation of hybrid performance. GCA: general combining ability, and SCA: specific combining ability.

Trait	Effect	GCA	GCA+SCA
DTA	(Intercept)	73.039659	73.050126
	Year: 2020	7.021365	7.009346
	Female_CUSo: CUSo09108	-4.67719	-4.679576
PH	(Intercept)	169.64751	169.336022
	Year: 2020	-10.02736	-10.196128
	Female_CUSo: CUSo09108	-2.106451	-1.583089
TGW	(Intercept)	24.256367	24.259181
	Year: 2020	-5.90046	-5.90689
	Female_CUSo: CUSo09108	1.724504	1.704036
GY	(Intercept)	50.211668	50.170341
	Year: 2020	-14.392578	-14.473595
	Female_CUSo: CUSo09108	-7.813219	-7.782964
GNP	(Intercept)	112258.499	112164.931
	Year: 2020	-24623.967	-24875.215
	Female_CUSo: CUSo09108	-20717.963	-20503.02

Table S6. Model fit for estimating general combining ability (GCA) of male lines using genomic information. SCA: specific combining ability, AIC: Aikake information criterion, BIC: Bayesian Information Criterion.

Trait	GCA model		GCA+SCA model	
	**AIC**	**BIC**	**AIC**	**BIC**
Days to anthesis	500.55	517.73	483.89	501.08
Plant height	415.02	432.26	179.61	196.84
Thousand grain weight	467.55	484.29	449.59	466.32
Grain yield	984.75	1000.77	976.97	992.99
Grain number per plot	1029.81	1045.75	1024.28	1040.22
